# Lymphatic Dissemination in Prostate Cancer: Features of the Transcriptomic Profile and Prognostic Models

**DOI:** 10.3390/ijms24032418

**Published:** 2023-01-26

**Authors:** Elena A. Pudova, Anastasiya A. Kobelyatskaya, Irina V. Katunina, Anastasiya V. Snezhkina, Maria S. Fedorova, Vladislav S. Pavlov, Ildar R. Bakhtogarimov, Margarita S. Lantsova, Sergey P. Kokin, Kirill M. Nyushko, Boris Ya. Alekseev, Dmitry V. Kalinin, Nataliya V. Melnikova, Alexey A. Dmitriev, George S. Krasnov, Anna V. Kudryavtseva

**Affiliations:** 1 Engelhardt Institute of Molecular Biology, Russian Academy of Sciences, 119991 Moscow, Russia; 2 National Medical Research Radiological Center, Ministry of Health of the Russian Federation, 125284 Moscow, Russia; 3 Vishnevsky Institute of Surgery, Ministry of Health of the Russian Federation, 117997 Moscow, Russia

**Keywords:** prostate cancer, lymphatic dissemination, prognosis, markers, RNA-Seq, genes, microRNAs, models, machine learning

## Abstract

Radical prostatectomy is the gold standard treatment for prostate cancer (PCa); however, it does not always completely cure PCa, and patients often experience a recurrence of the disease. In addition, the clinical and pathological parameters used to assess the prognosis and choose further tactics for treating a patient are insufficiently informative and need to be supplemented with new markers. In this study, we performed RNA-Seq of PCa tissue samples, aimed at identifying potential prognostic markers at the level of gene expression and miRNAs associated with one of the key signs of cancer aggressiveness—lymphatic dissemination. The relative expression of candidate markers was validated by quantitative PCR, including an independent sample of patients based on archival material. Statistically significant results, derived from an independent set of samples, were confirmed for miR-148a-3p and miR-615-3p, as well as for the *CST2*, *OCLN*, and *PCAT4* genes. Considering the obtained validation data, we also analyzed the predictive value of models based on various combinations of identified markers using algorithms based on machine learning. The highest predictive potential was shown for the “CST2 + OCLN + pT” model (AUC = 0.863) based on the CatBoost Classifier algorithm.

## 1. Introduction

Prostate cancer (PCa) is one of the most common cancers in men worldwide; more than one million new cases are diagnosed annually [1]. Prostate cancer is characterized by high clinical heterogeneity, which is manifested by a different propensity for recurrence of the disease, as well as the onset of progression after surgical treatment. The clinical heterogeneity of PCa, in turn, is due to molecular heterogeneity—in the tumors of patients, there are disorders that are accompanied by various changes in signaling pathways and metabolic processes [2,3]. At the same time, for the development of aggressive forms of PCa, the occurrence of only a few driver disorders is sufficient [4,5,6].

Despite a wide range of therapeutic approaches, the problem of choosing the tactics for treating a patient after radical prostatectomy is quite acute, especially for the category of patients with locally advanced PCa (LAPC). In addition to invasion of the prostatic capsule, metastasis to regional lymph nodes is often observed in LAPC. In this case, as a rule, the choice is between the immediate start of adjuvant hormone therapy or active monitoring of the level of prostate-specific antigen (PSA) in the blood.

The choice of a therapeutic concept is based on determining the prognosis for the patient, which, in turn, is based on such clinical and pathological parameters as the PSA level, the size of the primary tumor (stage T), and the Gleason score. Indeed, it is worth noting the emergence of an improved classification of cancer cell differentiation based on the Gleason score, a system developed by the ISUP (International Society of Uropathologists) [7]. The division into groups occurs between 1 and 5, depending on the Gleason score, the method providing the most accurate stratification of tumors.

However, these indicators are not informative enough to identify a group of patients with tumors that have high potential for progression, the development of an aggressive phenotype, and metastasis [8,9]. Thus, reliable prognostic markers are needed, the use of which, in combination with clinicopathological parameters, will help to reliably identify an aggressive tumor phenotype and thus choose the best therapeutic approach for the patient. One of the promising approaches in the search for prognostic markers may be the analysis of the transcriptomic data of tumors [10,11,12]. Currently, several prognostic expression panels of PCa markers based on tissue analysis have been identified, such as Decipher and Oncotype DX Genomic Prostate Score (GPS).

The Decipher test measures the RNA expression levels of 22 different genes, selected based on unique differential expression patterns in early metastasis [13]. The Decipher test has shown high discrimination in the prediction of clinical metastases (AUC = 0.75–0.83) and mortality from PCa (AUC = 0.78) in validation studies, significantly exceeding the available clinicopathological characteristics (AUC = 0.69) [14]. The Oncotype DX panel is a tissue biopsy-based genomic assay that measures the mRNA expression of 17 genes responsible for tumor cell growth and survival [15]. At the development stage of this test, we used the results of quantitative PCR, based on the archival material of tissue samples obtained after the surgical treatment of patients with PCa in low- and intermediate-risk groups. Based on validation studies, the Oncotype DX panel has been shown to be highly correlated with the biochemical recurrence of PCa and a poor prognosis, highlighting its predictive value for patients with PCa in low- and intermediate-risk groups.

Previously, we conducted a study that included miRNA-Seq analysis of 44 PCa tissue samples with and without lymphatic dissemination (N1 group = 20 samples; N0 group = 24 samples), as a result of which we identified a number of miRNAs, the expression of which could potentially be associated with lymphatic dissemination [16]. In the present study, we performed RNA-Seq profiling of an expanded sample of 73 PCa tissue samples from Russian patients and validated the obtained results by quantitative PCR (qPCR). This study also included an independent sample of 37 PCa tissue samples based on archival material. The obtained qPCR data for both PCa samples were used to analyze the predictive value of models based on combinations of candidate markers and clinicopathological parameters, using various machine learning algorithms (Logistic Regression (LR; scikit-learn ver.1.1.3), Light Gradient Boosting Machine (LGBM; ver.3.3.2), CatBoost (ver.1.0.6), Random Forest (scikit-learn ver.1.1.3) and XGBoost (ver.1.6.2)). The results of the study can be used to develop an expression panel for assessing the metastatic potential of high-risk PCa when choosing a therapeutic concept for a patient.

## 2. Results

### 2.1. Transcriptome Profile Associated with Lymphatic Dissemination

**Figure 1:**
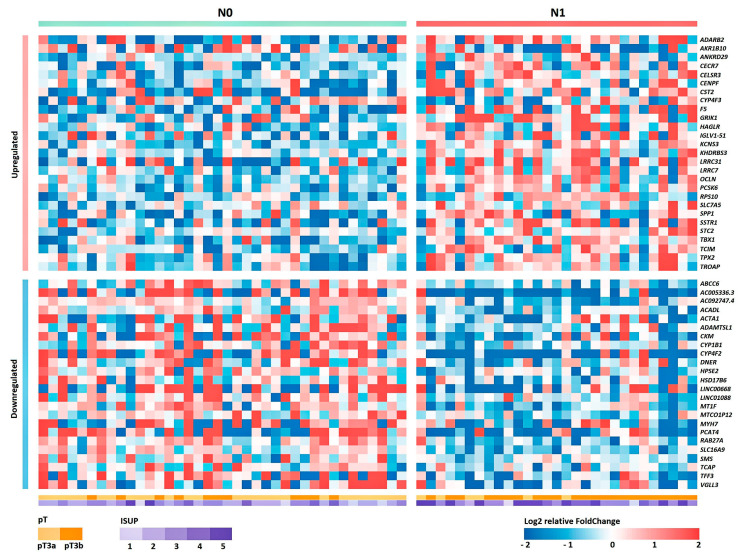
Heat map of the relative level of expression of 50 DEGs between groups N0 and N1 for Russian patients with LAPC. Cell colors correspond to the binary logarithm of the ratio of the expression level in the current sample to the average level for all samples (for each gene).

Based on the obtained RNA-Seq data, the differential expression (DE) of genes was analyzed between groups of patients with and without lymphatic dissemination (N1 and N0 groups, respectively). The list of obtained statistically significant DE genes (DEGs) is presented in Table S1 (*p* value of the QLF/U tests < 0.05). A heat map of the gene expression profile is shown in Figure 1.

Based on the obtained list of DEGs, pathway enrichment analysis was performed based on the GSEA algorithm using the Reactome 2022 database (Table 1).

According to the results of the pathway enrichment analysis, in the case of tumor samples of the N1 group, we predominantly observed the activation of the cell cycle and translation pathways. It is also worth noting the decrease in the activity of the pathway—"Fatty acid metabolism.”

### 2.2. Selection of Candidate Markers of Lymphatic Dissemination

One of the key objectives of this study was the identification of promising markers of lymphatic dissemination. To solve this problem, the resulting list of DEGs was filtered based on the following parameters: FDR U-test < 0.05; −1 < LogFC < 1; *p* value r_s_ < 0.05. As a result of the filtering, the following genes were selected that best match the specified criteria: *OCLN*, *F5*, *TBX1*, *CST2*, *RAB27A*, *PCAT4*, and *VGLL3* (Table 2).

Based on the previously obtained list of miRNAs associated with the N1 group, candidates were also selected based on filtering by similar parameters [16]. As a result, the following were selected: miR-148a-3p (Log2FC = −1.69; LogCPM = 18.6; FDR U test = 4 × 10^−2^; *p* value *r_s_* = 4 × 10^−2^) and miR-615-3p (Log2FC = 1.32; LogCPM = 7.6; FDR U test = 9 × 10^−3^; *p* value *r_s_* = 8 × 10^−3^).

**Figure 2:**
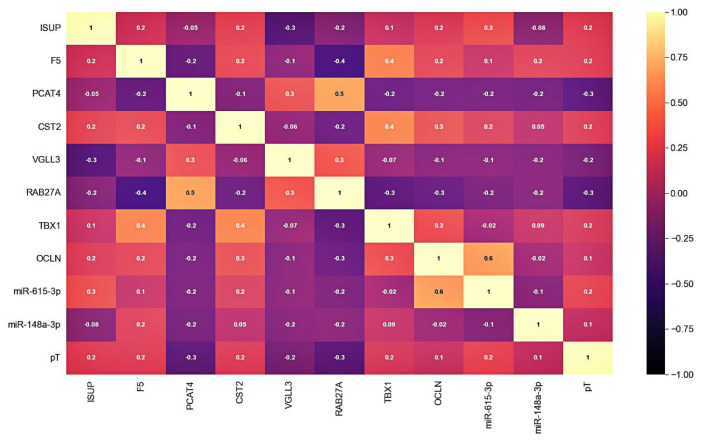
Correlation matrix of candidate markers and clinicopathological parameters expression levels. Cell colors correspond to the value of the Pearson correlation coefficient.

After selecting a number of candidate markers, we evaluated their multicollinearity, including those with clinical and pathological parameters—ISUP and pT stage. The analysis of the correlation showed that there is no functional strong relationship between the markers and that they can be jointly considered as part of the models (Figure 2).

### 2.3. Validation of the Relative Expression of the Potential Markers by qPCR

Validation of the expression of promising markers was primarily carried out on a previously sequenced sample of Russian patients with LAPC based on freshly frozen surgical material (FFT samples).

**Figure 3:**
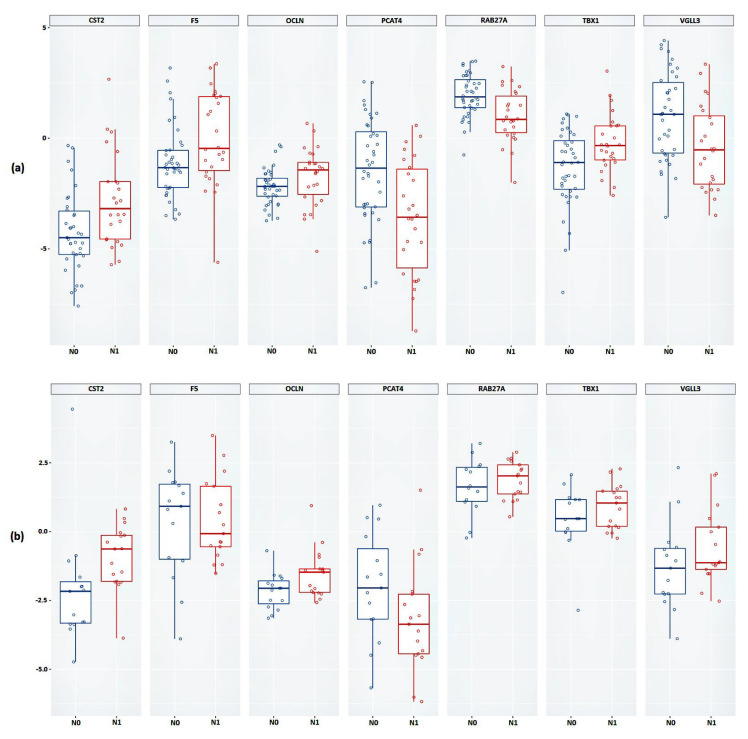
Box plot of the relative mRNA expression levels (in log scale) of *CST2*, *F5*, *OCLN*, *PCAT4*, *RAB27A*, *TBX1*, and *VGLL3* genes between N0 and N1 groups in (**a**) FFT samples, (**b**) FFPE samples. Group N0 is marked in blue, group N1 is marked in red.

As a result of the validation, it was shown that, based on the expression of all considered genes, a statistically significant difference between the studied groups was confirmed (Figure 3a and Table 3). Next, we validated the relative expression of the selected genes in an independent sample of Russian patients (FFPE samples).

Statistically significant results were obtained based on the relative expression of the *CST2*, *OCLN*, and *PCAT4* genes (Figure 3b and Table 3). The results of the calculations of the average change in the relative expression of the genes between the studied groups, performed based on the FFPE samples, showed the greatest decrease in expression in the presence of lymphatic dissemination for the *PCAT4* gene (a 1.64-fold decrease) and the greatest increase in expression was shown for the *CST2* gene (a 3.25-fold increase).

**Figure 4:**
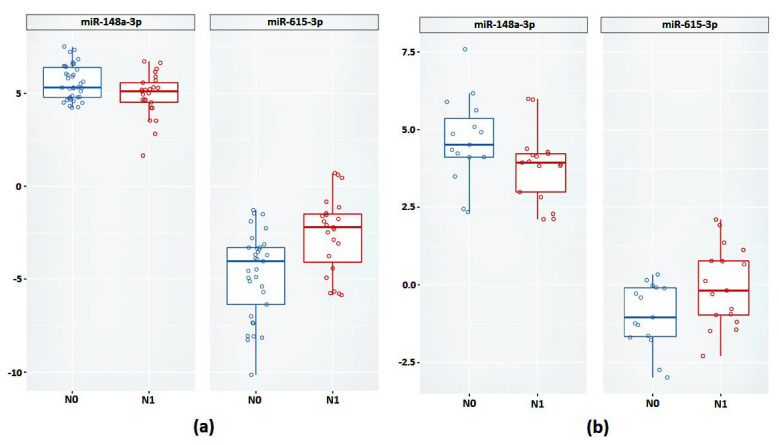
Relative expression of miR-148a-3p and miR-615-3p between groups N0 and N1 (**a**) on FFT samples (**b**) on FFPE samples.

Next, we validated the relative expression of the selected promising miRNAs. In the case of the FFT samples, a statistically significant difference between groups based on the relative expression of miR-148a-3p and miR-615-3p was confirmed (Figure 4a and Table 4).

Next, we validated the expression of miR-148a-3p and miR-615-3p in the FFPE samples. According to the results obtained, based on the relative expression of these miRNAs, there was also a statistically significant difference between the groups (Figure 4b).

In the case of miR-615-3p, we observed a 4.08-fold increase in expression in the case of the FFT samples (*p* = 0.001) and a 2.23-fold increase in the case of the FFPE samples (*p* = 0.04). The expression of miR-148a-3p was reduced in the N1 group by 1.5-fold in the FFPE samples (*p* = 0.04) and 2.04-fold in the FFPE samples (*p* = 0.04).

### 2.4. Relative Expression of the Candidate Markers in Lymph Node Metastases

We also analyzed the relative expression of the candidate markers in lymph node metastasis samples. Relative expression of *CST2*, *OCLN*, miR-148a-3p and miR-615-3p was found in all PCa metastasis samples.

**Figure 5:**
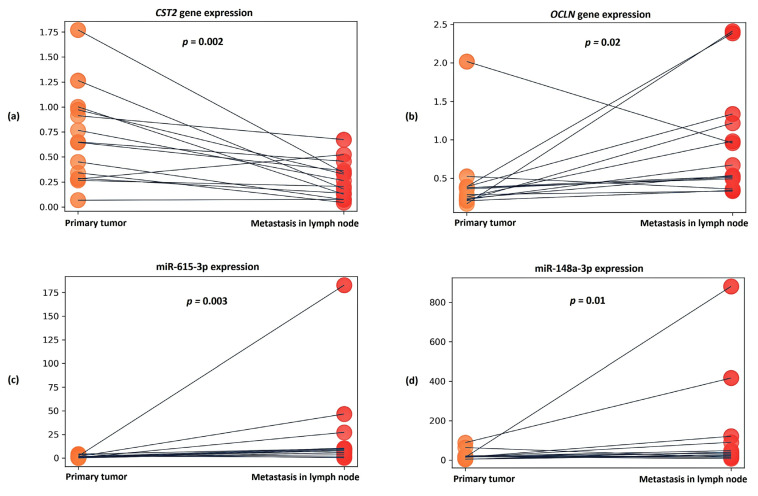
Dot plots showing relative expression results for (**a**) *CST2* gene, (**b**) *OCLN* gene, (**c**) miR-615-3p, and (**d**) miR-148a-3p between groups of primary tumors and lymph node metastases. The *y* axis represents the 2^−ΔCT^ values. The gray line indicates the direction of relative expression of the transcript between groups for each patient.

It was also found that in the metastasis samples, the expression of the CST2 gene was statistically significantly reduced by an average of 3.5-fold compared to the primary tumor samples (*p* = 0.002) (Figure 5a). In the case of the OCLN gene, in metastases, on the contrary, an average 3.34-fold increase in expression (*p* = 0.02) was observed (Figure 5b).

When evaluating the expression of candidate microRNAs in metastasis samples, a statistically significant increase in miR-615-3p expression was found—being, on average, 6.57-fold in comparison to primary tumor samples (*p* = 0.003) (Figure 5c). In the case of miR-148a-3p, an average 2.16-fold increase in expression (*p* = 0.01) was also noted in the metastatic samples (Figure 5d).

### 2.5. ROC–AUC Analysis of Models Based on Combinations of the Markers

We assessed the predictive value of the identified candidate markers in various combinations (from single predictors to a model based on all seven predictors). Each combination of predictors was evaluated based on five machine learning algorithms: Logistic Regression, LGBM, Catboost, Random Forest, and XGBoost. The results of all predictor combinations for the five algorithms are presented in Table S2.

Based on the results obtained in each category of combinations, we selected the best models in terms of the ROC–AUC value using the test data, which passed the threshold of 0.7 for the accuracy parameter in the training data (Table 4).

**Figure 6:**
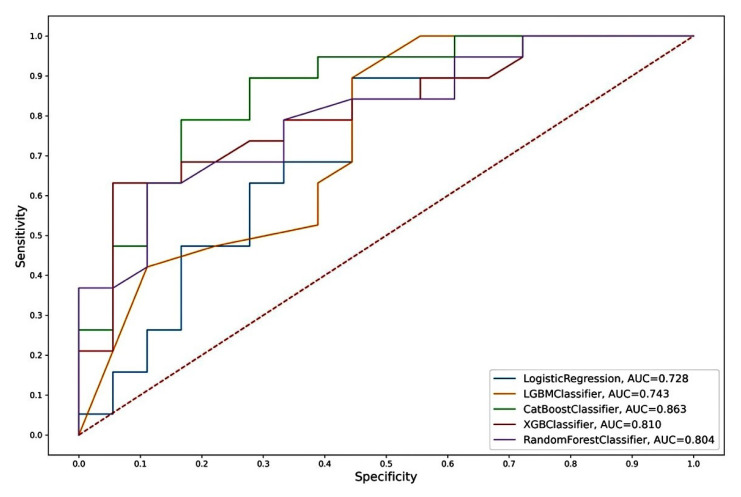
ROC curves of the “CST2 + OCLN + pT” model from Logistic Regression, LGBM, Catboost, Random Forest, and XGBoost algorithms.

The model based on the combination of «CST2+ OCLN+ pT» predictors and the CatBoost algorithm was chosen as the best one, characterized by the highest performance in all parameters considered. According to the other considered machine learning algorithms, we also observed high AUC values for this model (Table 5 and Figure 6).

## 3. Discussion

In the present study, a comprehensive transcriptomic analysis of LAPC tissue samples in a sample of Russian patients was carried out. This procedure aimed at identifying promising candidate markers of early metastasis. Based on the obtained transcriptomic profile, the biological pathways associated with lymphatic dissemination were first considered. The results of the biological pathways analysis with high enrichment predominantly demonstrated the activation of translational processes in tumor cells. This translational activity is likely to be closely related to increased metabolism, directed at obtaining energy from various sources to promote the epithelial–mesenchymal transition of tumor cells and metastasis.

Among all of the pathways identified, it is worth noting the decreased activation of the “Fatty Acid Metabolism” pathway. It is known that malignant transformation of a tumor depends on complex intercellular interactions, supported by a wide network of physical and chemical mediators that make up the tumor microenvironment [17]. Recently, various researchers have emphasized the key role of adipose tissue as a key component in the progression of solid tumors [18]. The prostate gland is surrounded by periprostatic adipose tissue, and extraprostatic expansion to adipose tissue is a widely recognized poor prognostic factor in PCa and an important predictor of recurrence after treatment [19]. The positive relationship between obesity and aggressive PCa, determined by an increase in local and distant spread, also supports the role of adipose tissue in tumor progression [20].

Interactions between adipocytes and tumor cells in the tumor microenvironment can create a metabolic symbiosis, leading to growth and metastasis. In combination with glucose, fatty acids are also vital for the synthesis of membrane lipids in tumor cells, energy production, and the synthesis of carcinogenesis-associated lipid-signaling molecules such as lysophosphatidic acids [21,22,23]. As a result, tumor cells activate de novo fatty acid synthesis, and elevated levels of fatty acid synthase are negatively correlated with prognosis [24]. Thus, in addition to synthesis, tumor cells can also use exogenous fatty acids as a source of nutrition. Thus, our results also highlight the importance of lipid metabolism in the progression of PCa.

Furthermore, based on the obtained transcriptomic profile, we searched for promising candidate markers based on gene expression. As a result of the validation, we confirmed the statistical significance of the expression of the *PCAT4*, *OCLN* and *CST2* genes in lymphatic dissemination in an independent sample.

The *PCAT4* (prostate cancer associated transcript 4; PCAN1; GDEP) gene is characterized by high tissue specificity for prostate tissue, but there are no published data on the biological function of this gene. According to the data obtained, we saw a significant decrease in the expression of this gene in the N1 group.

The *OCLN* (occludin) gene encodes the occludin protein, which belongs to tight junction proteins. Tight junctions are one of the key components in tumor metastasis, as tumor cells must pass through a series of barriers to successfully metastasize to secondary lesions [25]. OCLN is widely expressed in tissues and cells with tight junctions and is a membrane protein with four trans-membrane domains [26]. According to the literature, high expression of OCLN has been found in lung cancer, and when *OCLN* was knocked down in cell lines of lung cancer (A549, NCL-H1650, SPC-A1, HCC827, NCI-H1299, and MSTO-211H), inhibition of cell proliferation was observed in vitro and in vivo. In addition, *OCLN* knockdown promoted apoptosis of lung cancer cell lines and reduced their ability to invade, on the basis of which the role of *OCLN* as a tumor promoter and prometastatic factor was shown for the first time [27]. Based on our data, for the first time, an association between increased expression of the *OCLN* gene and the presence of lymphatic dissemination in LAPC was shown.

The *CST2* (cystatin SA) gene is a member of the cystatin family. Based on several studies, it has been shown that high expression of this gene is associated with the development of carcinogenesis. In breast cancer, increased expression of the *CST2* gene has been shown to be associated with tumor cell proliferation, movement, and adhesion [28]. Based on our data, we observed an association between increased expression of the *CST2* gene and lymphatic dissemination in LAPC.

In addition, we also validated previously identified promising markers based on miRNA expression, specifically miR-615-3p and miR-148a-3p, which also confirmed their association with lymphatic dissemination in the case of an independent sample of LAPC.

Aberrant expression of miR-615-3p has been described in many forms of cancer, including PCa, where overexpression of miR-615-3p has been observed in the most aggressive forms [29,30,31]. Experiments on cell lines of various types of cancer have shown that miR-615-3p overexpression supports cell proliferation and migration [29,30]. Functional studies performed on PCa cell lines have shown that miR-615-3p promotes proliferation, apoptosis, and migration of the PC3M cell line in vitro, indicating that miR-615-3p is an important oncogenic microRNA in PCa [31].

miR-148a-3p is one of the most highly expressed miRNAs in PCa tissues, as well as the most dominant in PCa metastasis [32]. High-grade tumors have been shown to exhibit reduced levels of miR-148a-3 expression. miR-148a expression has also been shown to be downregulated in docetaxel-resistant variants of PCa cell lines, including PC-3 and DU145, and downregulation of miR-148a has been observed in PCa with a risk of biochemical recurrence [33].

Evaluation of the expression of these candidate markers in samples of affected lymph nodes showed a further linear increase in the expression of the *OCLN* gene and miR-615-3p, as well as increased expression of miR-148a-3p. It can be assumed that the increased expression of these markers is not only associated with lymphatic dissemination in LAPC, but also supports the formation of secondary tumor foci.

We assessed the prognostic significance of *PCAT4*, *OCLN*, *CST2*, miR-615-3p, and miR-148a-3p for various combinations of predictors, both with each other and with such clinicopathological parameters as ISUP and pT. We considered the main metrics for models based on five machine learning algorithms for a classification problem.

The “CST2 + OCLN + pT” model, based on the CatBoost algorithm, had the highest metrics. In addition to the highest AUC (0.863), this model also had the highest sensitivity (SE = 0.83), specificity (SP = 0.79), and accuracy (AC = 0.81) of an independent sample of archival material. The parameters of this model based on other algorithms also differed in the highest rates (AUC = 0.73–0.81; SE = 0.56–0.78; SP = 0.68–0.84; AC = 0.62–0.73). Thus, the model we identified based on the predictor combination of «CST2 + OCLN + pT» had the highest prognostic potential for determining lymphatic dissemination in LAPC, both based on freshly frozen surgical material and, in the case of archival material, based on FFPE blocks.

## 4. Materials and Methods

### 4.1. Material

The present study included 73 samples of freshly frozen LAPC tissues, obtained as a result of radical prostatectomy with extended pelvic lymphadenectomy, a procedure performed on the basis of the research of National Medical Research Center for Radiology of the Ministry of Health of the Russian Federation.

The main criteria for sample inclusion in the study were the following: tumor type adenocarcinoma, LAPC (pT3a/3b), no neoadjuvant therapy, known lymph node status (N0/N1), and a negative resection margin for samples with stage N0. The sample of FFT samples was divided into groups, both with and without lymphatic dissemination (groups N1 *n* = 31 and N0 *n* = 42, respectively).

As an independent sample, 37 FFPE LAPC tissue samples were used, obtained as a result of radical prostatectomy with extended pelvic lymphadenectomy on the basis of the A.V. Vishnevsky National Medical Research Center for Surgery of the Ministry of Health of Russia. The sample was also divided into groups N1 (*n* = 19) and N0 (*n* = 18). Samples of affected regional lymph nodes were also included in the study of patients from group N1 (*n* = 14).

All samples of tumor tissues were characterized in the pathological anatomical departments of the respective medical institutions, on the basis of which the material was obtained, and they were found to contain at least 70% of tumor cells. The main clinical and pathological characteristics of patients are presented in Table 6.

### 4.2. Methods

#### 4.2.1. Isolation of Total RNA from Tissue Samples

Samples of fresh frozen tumor tissues were preliminarily homogenized using a MagNA Lyser device (Roche, Basel, Switzerland). Subsequent total RNA isolation was performed using the MagNA Pure Compact RNA Kit (Roche) on the MagNA Pure Compact System (Roche) according to the manufacturer’s protocol. For FFPE samples of tumor tissues and lymph nodes, total RNA isolation was performed using the High Pure FFPET RNA Isolation Kit (Roche) according to the manufacturer’s protocol. The concentration of isolated total RNA was assessed on a Quibit 4.0 fluorimeter (Thermo Fisher Scientific, Waltham, MA, USA) using the Qubit RNA BR Assay Kit (Thermo Fisher Scientific).

#### 4.2.2. Library Preparation and High Throughput Sequencing

For the obtained samples of total RNA, the quality was assessed on the Agilent Bioanalyzer 2100 instrument (gilent Technologies, Santa Clara, CA, USA) using the Agilent RNA 6000 Nano Kit (Agilent Technologies) in accordance with the manufacturer’s protocol. For the subsequent preparation of mRNA libraries, tumor tissue samples with a RIN value of at least 7 were used. Sample preparation of mRNA libraries was performed using the TruSeq Stranded mRNA Kit (Illumina, San Diego, CA, USA) in accordance with the manufacturer’s protocol. The concentration of the resulting libraries was measured on a Quibit 4.0 fluorimeter using the Qubit dsDNA HS Assay Kit (Thermo Fisher Scientific). The quality of the resulting libraries was assessed on an Agilent Bioanalyzer 2100 instrument using the Agilent High Sensitivity DNA Kit (Thermo Fisher Scientific) in accordance with the manufacturer’s protocol. The size of the resulting mRNA library was ~260 bp. High-throughput sequencing of mRNA libraries was performed on a NextSeq 500 System (Illumina) using NextSeq 500/550 High Output Kit v2.5 (Illumina) in 75 bp single-ended read mode. As a result of the sequencing, at least 14 million reads were obtained for each sample.

#### 4.2.3. Bioinformatics Data Analysis

For the obtained RNA-Seq data in the fastqc format, quality assessment was per-formed using the FastqQC and MultiQC programs (https://www.bioinformatics.babraham.ac.uk/projects/fastqc/, accessed on 10 May 2022). The Trimmomatic tool was used to remove adapter sequences from RNA-Seq data, which was followed by mapping to the reference genome (GRCh38 assembly) using the STAR tool [34,35]. FeatureCounts (Subread package v.1.6.4, Parkville, Australia) was used to calculate the read counts per gene [36]. The analysis of differential gene expression was performed in the R statistical environment using the edgeR package [37]. The TMM (Trimmed Mean of M-values) method was used to normalize the data. In the analysis of differential gene expression, the following quasi-likelihood F-test (QLF test) and the non-parametric Mann–Whitney test were applied (U-test). The Benjamini–Hochberg correction was applied to calculate the false positive rate (FDR). Spearman’s rank correlation coefficients (r_s_) were calculated between N0 and N1groups. Differences in the level of gene expression were considered statistically significant at test *p* values < 0.05. The visualization of heat maps of transcriptome profiles was performed using the ggplot2 package [38]. Biological pathway enrichment analysis based on RNA-Seq data was performed based on the GSEA algorithm using the Reactome 2022 database. The results were considered significant at FDR < 0.05.

#### 4.2.4. Quantitative PCR (qPCR)

cDNA samples were obtained from the mRNA template using Mint reverse transcriptase and oligo(dT) primer (20 µM) according to the protocol of the manufacturer (Evrogen, Moscow, Russia). cDNA was obtained from the miRNA template using the TaqMan Advanced miRNA cDNA Synthesis Kit (Thermo Fisher Scientific) according to the manufacturer’s protocol. qPCR was performed in three technical replicates on an Applied Biosystems 7500 instrument (Thermo Fisher Scientific). The *HPRT1* gene was used as a reference gene for analysis of relative mRNA expression. The sequences of primers used to validate markers based on mRNA expression are shown in Table 7. When validating microRNAs, miR-28-3p was used as a control. For the detection of control and target miRNAs, commercial sets of primers and probes, all contained on the TaqManTM Advanced miRNA Assay (Thermo Fisher Scientific), were used: 477814_mir (miR-148a-3p), 478175_mir (miR-615-3p), 477999_mir (miR-28-3p). The level of relative expression of genes and microRNA for each study group was calculated by the ΔCt method. Visualization and statistical analysis of expression results were performed using paired Wilcoxon tests in Jupyter Notebook, Python (ver. 3.6).

#### 4.2.5. Model Analysis

Based on the qPCR results, we used five algorithms for supervised machine learning, including Logistic Regression (LR), Light Gradient Boosting Machine (LGBM), CatBoost, Random Forest and XGBoost. These methods were implemented using scikit-learn, lightgbm, catboost, xgboost libraries in Jupyter Notebook, Python (ver. 3.6). The presence of lymphatic dissemination was used as a target. FFT samples were used as the train set, FFPE samples were used as the test set. The models were trained using cross-validation (cv = 5). Receiver operating characteristics (ROC) curves were used to compare the performance of these five algorithms.

## 5. Conclusions

We performed RNA-Seq profiling of 73 LAPC tissue samples, obtained from radical prostatectomy, with extended lymphectomy. Using bioinformatics analysis, enriched biological pathways associated with lymphatic dissemination in LAPC were identified in a sample of Russian patients, a group which can be further studied in the search for new potential therapeutic targets. Moreover, based on the bioinformatics analysis, we identified a number of genes and microRNAs, the expression of which can be considered potential prognostic markers. As a result of the validation of candidate markers by qPCR on an independent sample of patients, statistically significant results were confirmed for the *PCAT4*, *OCLN*, and *CST2* genes, as well as miR-615-3p and miR-148a-3p. Based on the qPCR data obtained, we analyzed the prognostic significance of various combinations of these candidate markers, including those with the clinicopathological parameters ISUP and pT, using various machine learning algorithms. As a result, we showed that the model, based on the combination of «CST2 + OCLN + pT», was characterized by the highest predictive value (AUC = 0.863) for determining lymphatic dissemination, both on samples of freshly frozen PCa tissues and on samples of archival material.

## Figures and Tables

**Table 1:** Enriched biological pathways, according to the Reactome 2022 database, associated with lymphatic dissemination in LAPC in a sample of Russian patients.

Pathway ID	Pathway Name	ES	NES	FDR	Gene Set Size	Matched Size
R-HSA-156842	Eukaryotic Translation Elongation	0.64	2.51	1 × 10^−3^	90	39
R-HSA-6791226	Major Pathway of rRNA Processing in Nucleolus and Cytosol	0.58	2.47	1 × 10^−3^	179	58
R-HSA-9633012	Response Of EIF2AK4 (GCN2) to Amino Acid Deficiency	0.62	2.41	1 × 10^−3^	98	41
R-HSA-72312	rRNA Processing	0.56	2.3	1 × 10^−3^	199	64
R-HSA-975956	Nonsense-Mediated Decay (NMD) Independent of Exon Junction Complex (EJC)	0.64	2.29	1 × 10^−3^	92	37
R-HSA-156902	Peptide Chain Elongation	0.64	2.26	1 × 10^−3^	86	37
R-HSA-8868773	rRNA Processing in Nucleus and Cytosol	0.56	2.22	1 × 10^−3^	189	62
R-HSA-168273	Influenza Viral RNA Transcription and Replication	0.57	2.21	1 × 10^−3^	137	47
R-HSA-192823	Viral mRNA Translation	0.61	2.21	1 × 10^−3^	90	38
R-HSA-69278	Cell Cycle Mitotic	0.47	2.1	3 × 10^−3^	523	124
R-HSA-72689	Formation of a Pool of Free 40S Subunits	0.58	2.1	3 × 10^−3^	98	44
R-HSA-975957	Nonsense-Mediated Decay (NMD) Enhanced by Exon Junction Complex (EJC)	0.56	2.1	3 × 10^−3^	112	42
R-HSA-2408557	Selenocysteine Synthesis	0.61	2.12	3 × 10^−3^	90	38
R-HSA-72764	Eukaryotic Translation Termination	0.64	2.13	3 × 10^−3^	90	37
R-HSA-156827	L13a-mediated Translational Silencing of Ceruloplasmin Expression	0.58	2.13	4 × 10^−3^	108	45
R-HSA-72737	Cap-dependent Translation Initiation	0.56	2.16	4 × 10^−3^	116	46
R-HSA-397014	Muscle Contraction	−0.61	−2.21	4 × 10^−3^	196	30
R-HSA-2408522	Selenoamino Acid Metabolism	0.55	2.03	1 × 10^−2^	114	41
R-HSA-72706	GTP Hydrolysis and Joining of 60S Ribosomal Subunit	0.56	2.01	1 × 10^−2^	109	46
R-HSA-1640170	Cell Cycle	0.45	1.99	1 × 10^−2^	654	142
R-HSA-168255	Influenza Infection	0.53	1.96	2 × 10^−2^	157	51
R-HSA-9010553	Regulation of Expression of SLITs and ROBOs	0.52	1.98	2 × 10^−2^	167	55
R-HSA-68877	Mitotic Prometaphase	0.52	1.91	3 × 10^−2^	186	41
R-HSA-1799339	SRP-dependent Cotranslational Protein Targeting to Membrane	0.49	1.87	3 × 10^−2^	108	43
R-HSA-68886	M Phase	0.44	1.89	3 × 10^−2^	380	86
R-HSA-2500257	Resolution of Sister Chromatid Cohesion	0.54	1.88	3 × 10^−2^	106	30
R-HSA-500792	GPCR Ligand Binding	0.53	1.9	3 × 10^−2^	458	35
R-HSA-9711097	Cellular Response to Starvation	0.48	1.86	3 × 10^−2^	153	50
R-HSA-8953854	Metabolism of RNA	0.41	1.9	3 × 10^−2^	666	156
R-HSA-446652	Interleukin-1 Family Signaling	0.51	1.84	4 × 10^−2^	152	30
R-HSA-68882	Mitotic Anaphase	0.47	1.83	4 × 10^−2^	232	65
R-HSA-72766	Translation	0.44	1.84	4 × 10^−2^	281	76
R-HSA-69620	Cell Cycle Checkpoints	0.45	1.84	4 × 10^−2^	271	69
R-HSA-9754678	SARS-CoV-2 Modulates Host Translation Machinery	0.61	1.82	4 × 10^−2^	47	17
R-HSA-141444	Unattached Kinetochores Signal Amplification Via A MAD2 Inhibitory Signal	0.5	1.81	4 × 10^−2^	93	28
R-HSA-69618	Mitotic Spindle Checkpoint	0.48	1.81	4 × 10^−2^	110	36
R-HSA-8978868	Fatty Acid Metabolism	−0.49	−1.95	4 × 10^−2^	173	32
R-HSA-72662	mRNA Activation Upon Binding of Cap-Binding Complex and eIFs Subsequent Binding to 43S	0.53	1.8	4 × 10^−2^	58	20

**Table 2:** Differential expression of promising genes as markers of lymphatic dissemination based on a sample of Russian patients with LAPC.

Gene ID	Symbol	Name	Log2FC	Log2CPM	FDR (U Test)	*r_s_*	*p* (r_s_)
ENSG00000197822	*OCLN*	Occludin	1.09	3.5	9 × 10^−3^	0.47	9 × 10^−4^
ENSG00000184058	*TBX1*	T-Box Transcription Factor 1	1.15	4.9	3 × 10^−3^	0.56	1 × 10^−5^
ENSG00000198734	*F5*	Coagulation Factor V	1.89	6	1 × 10^−2^	0.44	2 × 10^−3^
ENSG00000170369	*CST2*	Cystatin SA	1.71	2.7	1 × 10^−2^	0.47	9 × 10^−4^
ENSG00000069974	*RAB27A*	Ras-Related Protein Rab-27A	−1.17	6.5	5 × 10^−3^	−0.52	9 × 10^−5^
ENSG00000251321	*PCAT4*	Prostate Cancer-associated Transcript 4	−2.51	5.2	2 × 10^−2^	−0.4	9 × 10^−3^
ENSG00000206538	*VGLL3*	Vestigial-like Family Member 3	−2.16	5.1	3 × 10^−2^	−0.37	2 × 10^−3^

**Table 3:** The results of the relative expression of *CST2*, *F5*, *OCLN*, *PCAT4*, *RAB27A*, *TBX1*, and *VGLL3* genes between groups N0 and N1 based on the U test.

Gene	FC, FFT	*p* Value (U Test), *FFT*	FC, FFPE	*p* Value (U Test), *FFPE*
*PCAT4*	↓ 3.73	1 × 10^−3^	↓ 1.64	4 × 10^−2^
*OCLN*	↑ 1.63	1 × 10^−2^	↑ 1.77	4 × 10^−2^
*F5*	↑ 2.46	6 × 10^−3^	↓ 1.01	0.8
*CST2*	↑ 3.89	4 × 10^−3^	↑ 3.24	2 × 10^−3^
*RAB27A*	↓ 1.72	1 × 10^−3^	↑ 1.05	0.6
*TBX1*	↑ 2	1 × 10^−2^	↑ 1.23	0.3
*VGLL3*	↓ 3.5	6 × 10^−3^	↑ 1.35	0.2

↑, increase in relative gene expression; ↓, decrease in relative gene expression.

**Table 4:** The best models by AUC in each category of predictor combinations based on Logistic Regression, LGBM, Catboost, Random Forest, and XGBoost algorithms.

Model	Accuracy	AUC	F1	Sensitivity	Specificity
**Logistic Regression**					
CST2 + pT	0.65	0.79	0.72	0.39	0.89
ISUP + OCLN + pT	0.65	0.82	0.67	0.61	0.68
ISUP + PCAT4 + OCLN + pT	0.65	0.83	0.67	0.61	0.68
ISUP + PCAT4 + OCLN + miR148a + pT	0.65	0.83	0.67	0.61	0.68
ISUP + PCAT4 + CST2 + OCLN + miR148a + pT	0.65	0.83	0.67	0.61	0.68
**LGBM**					
OCLN + pT	0.62	0.70	0.63	0.61	0.63
ISUP + OCLN + pT	0.73	0.76	0.74	0.72	0.74
ISUP + OCLN + miR148a + pT	0.73	0.81	0.74	0.72	0.74
ISUP + PCAT4 + CST2 + OCLN + pT	0.73	0.80	0.74	0.72	0.74
PCAT4 + CST2 + OCLN + miR148a + miR615 + pT	0.59	0.75	0.69	0.28	0.89
**Catboost**					
miR615 + pT	0.68	0.71	0.74	0.44	0.89
CST2 + OCLN + pT	0.81	0.86	0.81	0.83	0.79
ISUP + OCLN + miR148a + pT	0.78	0.86	0.79	0.78	0.79
ISUP + PCAT4 + OCLN + miR148a + pT	0.70	0.82	0.70	0.72	0.68
ISUP + PCAT4 + CST2 + OCLN + miR148a + pT	0.70	0.80	0.72	0.67	0.68
**Random Forest**					
ISUP + miR615	0.59	0.65	0.63	0.50	0.68
OCLN + miR615 + pT	0.70	0.76	0.73	0.61	0.79
ISUP + OCLN + miR615 + pT	0.68	0.77	0.73	0.50	0.84
PCAT4 + OCLN + miR148a + miR615 + pT	0.65	0.77	0.72	0.39	0.89
ISUP + PCAT4 + OCLN + miR148a + miR615 + pT	0.70	0.76	0.74	0.56	0.84
**XGBoost**					
ISUP + miR615	0.57	0.63	0.60	0.50	0.63
OCLN + miR615 + pT	0.76	0.76	0.78	0.67	0.84
ISUP + OCLN + miR615 + pT	0.70	0.78	0.72	0.67	0.74
ISUP + OCLN + miR148a + miR615 + pT	0.68	0.76	0.71	0.56	0.79
ISUP + PCAT4 + OCLN + miR148a + miR615 + pT	0.68	0.76	0.71	0.56	0.79

**Table 5:** ROC analysis results for the «CST2+ OCLN+ pT» model based on the Logistic Regression, LGBM, Catboost, Random Forest, and XGBoost algorithms.

Metrics	Logistic Regression	LGBM	Catboost	Random Forest	XGBoost
Accuracy, Train	0.76	0.72	0.70	0.69	0.67
AUC, Train	0.84	0.88	0.98	1.00	1.00
F1, Train	0.70	0.63	0.63	0.62	0.61
Accuracy, Test	0.70	0.62	0.81	0.73	0.73
AUC, Test	0.73	0.74	0.86	0.80	0.81
F1, Test	0.74	0.65	0.81	0.72	0.74
Sensitivity, Test	0.56	0.56	0.83	0.78	0.72
Specificity, Test	0.84	0.68	0.79	0.68	0.74

**Table 6:** Clinicopathological characteristics of LAPC specimens from two cohorts of Russian patients.

	FFT Samples		FFPE Samples	
Group	N0	N1	N0	N1
Age	46–77 (63)	41–73 (63)	56–75 (65)	51–72 (65)
pT3a stage	29	7	8	2
pT3b stage	13	24	10	17
ISUP group 1	5	2	5	1
ISUP group 2	17	2	4	5
ISUP group 3	12	11	4	2
ISUP group 4	5	5	4	2
ISUP group 5	3	11	1	9
PSA, ng/ml	3.4–27.6 (14.81)	5.49–44 (14.28)	7.7–46 (14.6)	4–20 (12.9)

**Table 7:** Primer sequences for detection of marker gene expression by qPCR.

Gene	Primer Sequence (5′→3′)	Product Size, bp
*HPRT1*	F: CGAGATGTGATGAAGGAGATGG	98
	R: TTGATGTAATCCAGCAGGTCAG	
*F5*	F: TCTTACCTTGACCACACATTCC	99
	R: TCCACTGTCCTCACTGATACT	
*PCAT4*	F: GGATTCGCAAGAGAACACAATC	111
	R: CATCACAAACCGGCCAATATC	
*OCLN*	F: GGTTCACTTCTCCCAGTCTTTC	96
	R:AGACACAATCAACAGGGTTAGG	
*RAB27A*	F: CATGCCTGGGATCTTCTCTATG	111
	R: CCGGATGCTTTATTCGTAGGT	
*TBX1*	F: CGACAACGGCCACATTATTC	100
	R: CTCGGCATATTTCTCGCTATCT	
*CST2*	F: AGGAGGACAGGATAATCGAGG	84
	R: TGATGACAAAGTGAAGGGCAC	
*VGLL3*	F: CTCTCAAGCCAGCGGAATAG	102
	R: GACCTGGAAGTCAGGATGAAC	

## Data Availability

All data generated or analyzed during this study are available within the article or upon request from the corresponding author.

## Supplementary Materials

The following supporting information can be downloaded at: https://www.mdpi.com/article/10.3390/ijms24032418/s1.
